# Engagement and predictors of use of a smartphone app for migraine self‐management: A secondary analysis of the EMMA trial

**DOI:** 10.1111/head.70009

**Published:** 2025-11-25

**Authors:** Stefanie Lysk, Daniel Pach, Tatjana Tissen‐Diabaté, Simon Scholler, Claudia M. Witt

**Affiliations:** ^1^ Institute of Social Medicine, Epidemiology and Health Economics Charité—Universitätsmedizin Berlin, Corporate Member of Freie Universität Berlin and Humboldt‐Universität zu Berlin Berlin Germany; ^2^ Robert Koch Institute Berlin Germany; ^3^ Institute for Complementary and Integrative Medicine University Hospital Zurich and University Zurich Zurich Switzerland

**Keywords:** digital health, headache tracking, mHealth, migraine app, migraine management, user engagement

## Abstract

**Objective:**

The objective of this secondary analysis was to examine engagement with a mobile app for migraine self‐management and to identify predictors of migraine app usage.

**Background:**

Migraine self‐monitoring and behavioral strategies are central components in migraine self‐management, but patient adherence often remains a challenge. Recently, migraine apps have shown promise in enabling more frequent and continuous headache tracking and supporting self‐management by integrating behavioral strategies; however, data on patient engagement with these digital interventions are still scarce.

**Methods:**

This secondary analysis of the EMMA trial used the full dataset of the intervention group, which received a migraine app for 24 weeks, and examined app engagement and factors associated with frequent app usage. Outcomes were frequency of app engagement, intensity of app engagement, and time of usage. Potential associated factors included demographics, migraine history, burden of disease, headache management self‐efficacy, and first‐week app use. Descriptive analyses summarized engagement patterns for the overall app, the diary, and self‐management modules. Kaplan–Meier survival curves illustrated usage over time. Univariable linear regression models explored associations between patient characteristics and first‐week app usage as predictors of total app usage.

**Results:**

Of the 238 patients, 161 (67.7%) were still actively using the app after 24 weeks. Total active usage days ranged from 1 to 168, with a mean (SD) of 129.7 (52.5) days, corresponding to usage on approximately 77% of days. Among all app modules, the headache and trigger diaries had the highest usage frequencies, with means (SDs) of 110.6 (49.7) and 118.1 (60.9) days, respectively.

Engagement with the behavioral self‐management modules was lower overall, with faster drop‐off rates. Among the behavioral self‐management modules, the training module showed the highest usage, with a mean (SD) of 26.2 (33.9) documented training days. Linear regression analyses showed that older age and higher app usage during the first week were associated with more frequent app usage.

**Conclusions:**

This secondary analysis demonstrated high engagement with the headache and trigger diary modules of a migraine app over 6 months. These findings support the potential of smartphone apps to improve adherence to headache self‐monitoring. However, engagement with the behavioral self‐management modules was lower than intended, highlighting the persistent challenge of promoting adherence to behavioral migraine interventions.

**Trial Registration:**

German Clinical Trials Register: DRKS00024174

## Plain Language Summary

Many people with migraine rely on mobile apps to help track their headaches, triggers, and symptoms. This study examined how people with migraine used a self‐management app over 6 months and found that most users frequently tracked their headaches and triggers in the app's diary, but they used the behavioral self‐management tools less often and engagement with these features decreased over time. People who were older or who used the app more during the first week tended to continue using it longer overall.

**Abbreviations**
BCT: behavior change techniques
ICHD‐3: The International Classification of Headache Disorders, 3rd edition
mHealth: mobile Health

## BACKGROUND

Preventive migraine management is comprehensive and, in addition to pharmacological treatments, includes self‐management activities. Central components of self‐management are self‐monitoring and behavioral interventions. In clinical practice, maintaining a headache diary is a widely used method of self‐monitoring.^1^ It enables patients and clinicians to monitor the frequency and patterns of headache attacks and to evaluate treatment outcomes. For behavioral self‐management, clinical practice guidelines explicitly recommend biofeedback, relaxation training, and elements of cognitive behavioral therapy as Level A treatments.^2^, ^3^, ^4^, ^5^ A recent meta‐analysis of behavioral migraine interventions, which included 50 randomized controlled trials with a total of 6024 adults, found that most behavioral interventions are multimodal and combine several therapeutic components.^6^ The authors concluded that interventions incorporating relaxation training, mindfulness‐based stress reduction, or cognitive behavioral therapy can reduce attack frequency, although the overall strength of evidence remains low. In addition to these strategies, aerobic exercise has also been shown to be an effective self‐management intervention and is recommended in current clinical guidelines.^2^, ^3^, ^7^, ^8^

Another key migraine self‐management approach is trigger management. Although migraine triggers are highly individualized, several common triggers, such as inadequate hydration, irregular meals, stress, and poor sleep hygiene, have been consistently identified across studies.^9^, ^10^, ^11^, ^12^, ^13^ In clinical practice, recognition of individual patterns is typically supported by the use of headache und trigger diaries. Upon identifying individual triggers as well as protective factors, it is recommended to address them through targeted lifestyle modifications, with a focus on trigger management and not avoidance.^2^, ^14^

Despite clinical recommendations for these different non‐pharmacological self‐management approaches, patient engagement remains challenging.^11^, ^15^, ^16^, ^17^ A systematic review on treatment adherence in patients with headache found that adherence rates to behavioral lifestyle recommendations vary widely across studies, with long‐term adherence often being suboptimal.^18^ Unfortunately, only a minority of studies on behavioral interventions for migraine explicitly report adherence rates.^19^ Studies analyzing adherence to headache diary use have shown mixed results.^20^, ^21^

In recent years, there has been growing interest in the digitalization of migraine care, particularly with the rapid development of smartphone‐based applications and remote headache monitoring.^22^, ^23^, ^24^ Publicly available migraine apps primarily function as electronic headache and trigger diaries and are expected to improve diary adherence and tracking quality, support diagnosis, help identify individual triggers, and even predict migraine attacks.^22^, ^25^, ^26^ Some apps offer exportable reports that can be integrated into electronic health records or downloaded by patients. These features hold promise for improving patient–provider communication and enhancing diagnostic accuracy.^25^, ^27^, ^28^ Furthermore, studies show that electronic headache self‐monitoring can improve medication adherence and help prevent medication overuse.^29^, ^30^

Just like the app examined in this study, a smaller number of migraine management apps go beyond electronic headache diaries and facilitate access to behavioral self‐management approaches such as relaxation techniques or biofeedback.^26^ By integrating evidence‐based strategies designed to promote health behaviors, so‐called behavior change techniques (BCTs), these apps have the potential to activate migraine self‐management behavior and enhance long‐term engagement with migraine self‐management;^27^ however, current findings on the effectiveness of digital migraine interventions remain heterogeneous. Similarly, in the context of mobile Health (mHealth) solutions for other chronic conditions, the effectiveness of comprehensive therapeutic apps remains inconclusive. One commonly discussed reason for limited effectiveness is insufficient engagement: irregular use and early discontinuation are widespread challenges.^31^, ^32^, ^33^, ^34^ Unfortunately, systematic investigation of user engagement is often lacking in digital intervention studies.^34^, ^35^, ^36^, ^37^, ^38^

To date, no study has specifically examined engagement with a comprehensive, multimodal, smartphone‐delivered migraine self‐management intervention. Therefore, we aimed to (1) examine engagement with a smartphone app for migraine self‐management and (2) identify predictors of migraine app usage. We hypothesized that engagement would differ across the various app modules, that overall app use would decline over time, and that participant characteristics such as age and disease duration would influence patterns of usage.

## METHODS

### Study design

EMMA was a fully remote, two‐arm, open‐label, parallel‐group randomized controlled mHealth trial (German Clinical Trials Register: DRKS00024174). The trial received ethical approval from the Charité—Universitätsmedizin Berlin ethics committee (reference EA1/343/20). The study took place in Germany from March 2021 to February 2022 and evaluated the effectiveness of the digital health application M‐sense Migräne, a DiGA (Digitale Gesundheitsanwendung)^39^ that could be prescribed and reimbursed in Germany. Details of the study design and main results have been reported recently.^40^ Briefly, pre‐screened patients provided electronic in‐app informed consent and completed an electronic headache diary during a four‐week baseline period. After this run‐in phase, eligible patients were randomized in a 1:1 ratio to either the intervention group, which received the M‐sense Migräne app for 24 weeks, or the control group, which only received access to a basic electronic headache diary. The primary endpoint was the number of migraine days per month (month defined as 28 days) after 12 weeks, measured via the in‐app headache diary. For the present secondary analysis, only app usage data from participants in the intervention group were included.

### Participants

EMMA included adult patients diagnosed with episodic or chronic migraine, based on the criteria of the International Classification of Headache Disorders, 3rd edition (ICHD‐3). Eligibility criteria are detailed in Table 1. Participants included in this secondary analysis were those randomized to the intervention group who received the app for 24 weeks.

**TABLE 1:** Inclusion and exclusion criteria of EMMA study.

*Inclusion criteria*
Adults aged ≥18 years
Migraine diagnosis (ICD‐10 G43, all subcodes; verified by study physician in video consultation according to the ICHD‐3)
Disease duration of at least 1 year
Disease onset before age of 50 years
At least 3 documented migraine attacks throughout 28‐day baseline phase in the app diary
App headache diary adherence of at least 3 days/week throughout 28‐day baseline phase
Own a smartphone and report smartphone literacy
Sufficient German language skills
*Exclusion criteria*
Planned pregnancy; pregnant and breastfeeding women
Medication‐overuse headache (validated by app medication diary entries during baseline phase, > 15 days with headache medication use)
Use of M‐sense Migräne or similar apps of at least 1 month during the last 12 months
Planned start of a new migraine treatment within the next 4 months
Simultaneous participation in another interventional study

Abbreviation: ICD‐10, International Classification of Diseases, 10th Revision; ICHD‐3, International Classification of Headache Disorder, 3rd Edition.

### Migraine app

#### Structure of the app

**FIGURE 1:**
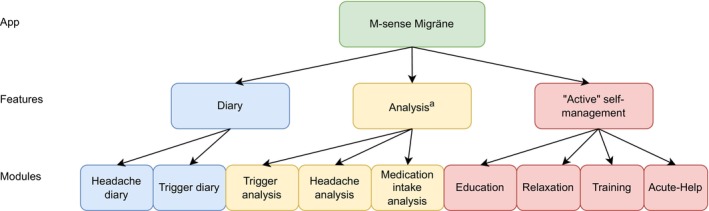
Hierarchical structure of the app illustrating main features and their associated app modules. ^a^The usage of the feature “Analysis” was not tracked by the app. [Color figure can be viewed at wileyonlinelibrary.com]

**FIGURE 2:**
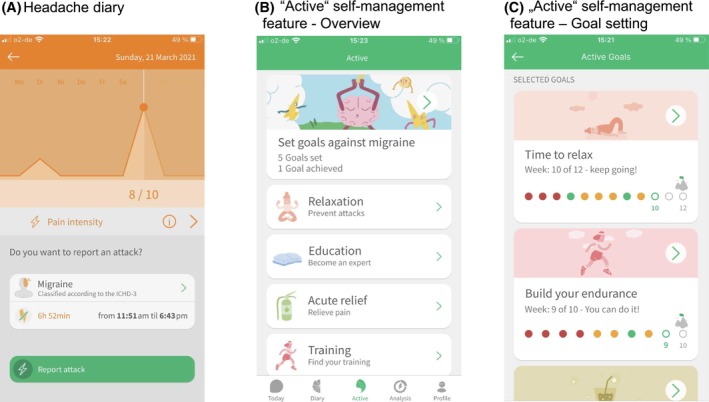
App screenshots of the M‐sense Migräne app. (A) Screenshot of the headache diary with a weekly overview of pain intensity. (B) Overview screen of the “Active” self‐management feature with the selectable modules Education, Relaxation, Acute‐help, and Training, along with the personal behavioral goal‐setting component. (C) Goal‐setting screenshot within the “Active” self‐management feature showing the Relaxation goal and Training goal. [Color figure can be viewed at wileyonlinelibrary.com]

The M‐sense Migräne app was classified as a Class I medical device under the Medical Device Directive. It was one of the most widely used headache apps in Germany.^24^, ^41^ The app consisted of three main features: (1) diary for tracking headaches, medication, and potential triggers; (2) data analysis feature; and (3) “Active” self‐management feature. The structure of the app is illustrated in Figure 1. Screenshots of the app are shown in Figure 2. Although the app included a physician report and an export function, it could be used independently without involvement of healthcare providers.

#### Diary

In the headache diary module (see screenshot A in Figure 2), patients were able to record pain intensity, quality, and localization, as well as accompanying symptoms (e.g., vomiting, photophobia) and headache medication intake. The app included an integrated, validated algorithm that automatically classified each reported headache episode according to the ICHD‐3 criteria, based on the entered symptoms.^42^ Additionally, the home screen featured a daily headache question that prompted patients to indicate whether or not they had experienced a headache on a given day. The purpose of this prompt was to document headache‐free days as well, in order to ensure the accuracy of the app's analysis feature. Within the trigger diary, patients could enter personal factors of interest or select from a predefined list of 12 common trigger and protective factors (e.g., water intake, sleep quality, stress level).

#### Analysis feature

The app provided descriptive analyses and visualizations of headache patterns and medication intake. It also issued automated warnings in cases of pain medication overuse. The trigger analysis offered a descriptive evaluation of tracked triggers. Patients were able to download a monthly physician report as a PDF, which included an attack calendar with automatically classified headache attacks and summary statistics.

#### “Active” self‐management feature

The “Active” feature focused on behavioral self‐management and included educational lessons, relaxation training, training guidance, and an acute‐help module, see screenshot B in Figure 2. The education module featured 33 knowledge lessons, delivered via a chatbot and tailored to the user's diary entries. The content of the lessons consisted of headache‐related knowledge, self‐management strategies and skills, as well as elements of cognitive behavioral therapy. Within the relaxation module, the app offered a range of audio recordings for relaxation techniques, including progressive muscle relaxation, breathing meditation, and guided imagery. The training module provided instructions and training plans for endurance sports. The acute‐help module included audio files for self‐guided imagery and instructional videos for temple and neck massage.

#### App‐based techniques to promote engagement

To support engagement with the different app modules, several adherence‐promoting elements were incorporated into the migraine app. A rule‐based chatbot named *Brainy* guided participants through the app and provided personalized feedback. At the beginning of diary use, the chatbot highlighted the clinical importance of tracking and helped participants set up a personalized diary, for example by selecting individual trigger factors to monitor. Participants were incentivized to track consistently by being informed that the analysis feature would only yield valid insights (e.g., trigger analysis) once at least 60 consecutive days of diary data had been entered. A streak bar displayed daily tracking continuity, further encouraging adherence. Tracking data were also summarized in a monthly physician report (PDF) that participants could download. In addition, visual feedback, calendars, reminders, and push notifications were used to maintain regular engagement.

For the self‐management feature, the chatbot introduced the clinical relevance of endurance sports, relaxation training, and education prior to the first use. Importantly, participants were not prescribed a specific self‐management strategy but were encouraged to try different options and choose what worked best for them (free‐choice approach). For example, several relaxation techniques were available, and participants were advised to experiment with the formats they preferred. The app also incorporated structured goal‐setting techniques (see screenshot C in Figure 2). Predefined goals included, for instance, relaxation training three times per week for 10 weeks, or endurance training three times per week for 12 weeks. Streak bars and reward mechanisms were also implemented within the education, relaxation, and training modules. Notably, all self‐management modules were designed to function as stand‐alone modules, allowing patients to engage with them independently according to their preferences.

The list of all 23 BCTs used in the app, categorized according to the Behavior Change Techniques Ontology vs. 1 by Marques et al.^43^ can be found in the Supporting Information Additional File 1.

### Outcome measures

Details on the primary and all secondary outcomes of the EMMA trial have been published elsewhere.^40^ This secondary analysis focuses on app engagement and analyses the usage log data collected by the migraine app.

In accordance with the FITT principle (Frequency, Intensity, Time, and Type)^35^, app engagement is evaluated using the following endpoints: frequency of engagement, intensity of engagement, and usage time (usage span). Frequency of engagement refers to how often the app or a module was used. Intensity reflects the depth of engagement, for example, the number of exercises completed. Time of engagement refers to the usage span or duration. To capture this, we calculated usage stickiness (defined as number of days between app activation and the last performed action) and usage persistence (defined as number of days until the first longer break in app use lasting at least 7 days). All endpoints are calculated for the following types: the overall app, the app features diary and “Active” self‐management, and in more granular detail for the six app modules (headache diary, trigger diary, education, relaxation exercises, training module, and acute help module). Usage data were not available for the analysis feature, as use of these sections was not logged by the app.

All measurements for app usage were documented daily. However, for the purpose of outcome calculation, data were aggregated across standardized 28‐day months and across the study period of 6 months (24 weeks). All outcomes are displayed in Table 2.

**TABLE 2:** App engagement outcomes mapped to the categories of frequency, intensity, and time of engagement, following the FITT principle.

FITT metric	Outcome	Overall app	Features (diary and “Active” self‐management)	App modules^a^
Frequency of engagement	Total active days over 24 weeks	×	×	×
	Monthly active days (month 1–month 6)	×	×	×
	User Activity Ratio^b^	×	×	×
Intensity of engagement	Average number of daily tracked triggers over 24 weeks^c^			× (trigger diary)
	Total number of exercises/lessons completed over 24 weeks			× (education, relaxation, training, acute help)
Time (usage span)	Stickiness^d^	×	×	×
	Persistence^e^	×	×	×

*Note*: Frequency refers to the number of usage days, intensity reflects the number of exercises or lessons completed, and time indicates the duration of app use.

Abbreviation: FITT principle, Frequency, Intensity, Time, and Type.

^a^ Headache diary, Trigger diary, Education module, Training module, Relaxation module, Acute‐help module.

^b^ User Activity Ratio is calculated as the number of total active days/number of study days (168).^50^

^c^ Twelve trigger factors could be tracked: water intake, mood, alcohol consumption, sleep duration, sleep quality, missed meals, stress level, smoking, caffeine consumption, energy level, activity level, and menstrual cycle.

^d^ Stickiness = number of days between app activation and the last performed action.

^e^ Persistence = number of days until the first longer break in app use lasting at least 7 days.

We defined the use of each specific module on a given day as follows:

- Headache diary: Documentation of headache symptoms or an active confirmation of the absence of symptoms by answering the daily headache question on the app's home screen.
- Trigger diary: Documentation of at least one trigger in the trigger diary
- Education module: Completion of a full chatbot lesson (starting a lesson without completion was not logged).
- Relaxation module: Either playing a relaxation audio file within the app or documenting the completion of a relaxation exercise performed outside the app.
- Acute help module: Playing an instructional video or a guided imagination audio file.
- Training module: Documentation of completed endurance training (usage of instructional content was not tracked).

Predictors analyzed for app usage frequency include app engagement in the first week and the following baseline variables from the EMMA trial: age, gender, education, disease duration (years since first manifestation), years since diagnosis, headache‐related disability (HIT‐6, 6‐item Headache Impact Test),^44^ headache‐attributed burden (HALT‐30, Headache Attributed Lost Time),^45^ and headache management self‐efficacy (HMSE‐G‐SF, Headache Management Self‐Efficacy Scale German Short Form).^46^

### Statistical analysis

For examining app engagement, we performed descriptive analyses (means and SDs, minimum and maximum values, medians, and interquartile ranges) for all outcome variables of Table 2 (frequency, intensity, and time). We analyzed the complete dataset and included all participants of the intervention group who received the app. A visual inspection of data distributions was carried out. Usage frequency was graphically displayed for the app, the two features diary and self‐management, and the six modules. To illustrate the usage time span, we generated Kaplan–Meier curves stratified by module.

The derived engagement variables are count data and, by definition, contain no missing values, as they are calculated daily with binary values of 0 (no entry/no usage) or 1 (usage). Log files were automatically generated whenever a participant actively engaged with a module (e.g., documenting symptoms, completing a chatbot lesson, playing an audio file). If the app was not used on a given day, no log was generated; therefore, the absence of a log entry indicates no usage rather than missing data, and such days were coded as 0. Even for usage intensity metrics, such as the number of completed exercises, the dataset does not contain missing values; on days without any entries or logged usage, the value is recorded as 0.

To examine predictors of app engagement, three key outcome measures were used: total active days of overall app use, total active days of diary use, and total active days of self‐management use. These outcomes were analyzed as continuous variables using linear regression models to explore associations with baseline patient characteristics and app usage frequency during the first week. Univariable linear regression models were used.

Effect estimates and 95% confidence intervals are presented to support interpretation of the magnitude and direction of associations. This analysis was intended as a preliminary inspection of associations, rather than the development of a predictive model. As this was an exploratory analysis, no formal significance level was defined, and all *p*‐values are interpreted in an exploratory manner. The results are intended to generate hypotheses and provide insight into potential associations between baseline characteristics and user engagement outcomes.

The details of the secondary engagement analysis were pre‐specified in Annex 2 of the statistical analysis plan (SAP) for the EMMA trial (see Supporting Information Additional File 2). All analysis were conducted using R language (version 4.4.2, R Foundation for Statistical Computing) and RStudio (Version 2024.12.0+467, date: 2024‐10‐31, Posit Software, PBC).

## RESULTS

### Participants

A total of 477 participants were enrolled in the EMMA trial, of whom 238 were allocated to the intervention group and included in this analysis. Within this group, 88.7% identified as women, and the mean (SD) age was 35.6 (10.3) years. The majority of participants (94.5%) had episodic migraine. The burden of migraine was high, as reflected by a mean (SD) HIT‐6 score of 64.2 (3.5) and a mean (SD) HALT‐30 score of 10.8 (8.2). All baseline characteristics are presented in Table 3.

**TABLE 3:** Baseline characteristics of the included patients from the intervention group in the EMMA trial (*n* = 235).

Parameter	All participants (*n* = 235)
	Mean (SD)/*n* (%)
Age	35.6 (10.3)
Gender	
Woman	211 (88.7)
Man	27 (11.3)
Gender diverse	0 (0)
Years since migraine onset	17.4 (10.7)
Years since migraine diagnosis	11.9 (9.0)
HIT‐6 baseline^a^	64.2 (3.5)
HALT‐30 baseline^b^	10.8 (8.2)
HSME‐G‐SF baseline^c^	24.2 (6.9)
Migraine type	
Episodic migraine	225 (94.5)
Chronic migraine	13 (5.5)
Education	
High School Diploma with university readiness	92 (38.7)
High School Diploma	46 (19.3)
Technical College Degree	39 (16.4)
Vocational training	4 (1.7)
Other	57 (23.9)

^a^ HIT‐6 = Headache Impact Test, score ranges from 36 to 78, a lower score indicates a lower disease burden.

^b^ HALT‐30 = Headache Attributed Lost Time, score ranges from 0 to 30, a lower score indicates less lost time.

^c^ HMSE‐G‐SF = Headache Management Self‐Efficacy Scale German Short Form, score ranges from 6 to 42, a lower score indicates less self‐efficacy.

### Results from the EMMA trial

The results of the EMMA trial have been reported elsewhere in detail.^40^ The migraine app did not demonstrate superiority in reducing the number of migraine days after 12 weeks of app use, compared to a basic electronic headache diary. Consistent with the primary outcome, most secondary endpoints also showed no differences between the intervention and control group.

### App engagement

#### Frequency of engagement

The main descriptive results for app usage frequency for the overall app and the app modules are presented in Table 4. All additional calculations are provided in the Supporting Information Additional File 3. During the first week, the app was used on a mean (SD) of 6.6 (1.0) days. The total number of active usage days over the 168‐day study period varied from 1 to 168, with a mean (SD) of 129.7 (52.5) total usage days, corresponding to usage on approximately 77% of days. However, the total number of active days per month decreased over time, from a mean (SD) 25.6 (5.3) monthly usage days in the first month to 18.0 (12.4) monthly usage days in month six.

**TABLE 4:** Engagement metrics for the overall app and individual modules, categorized according to the FITT principle.

Parameter	Overall app	Diary feature		“Active” self‐management feature			
		Headache diary	Trigger diary	Education module	Relaxation module	Training module	Acute help module
*Frequency of engagement (mean, SD)*							
Total active days over 24 weeks	129.7 (52.5)	110.6 (49.7)	118.1 (60.9)	3.4 (4.3)	19.0 (28.4)	26.2 (33.9)	2.6 (4.7)
Active days month 1^a^	25.6 (5.3)	23.6 (5.5)	22.6 (8.9)	2.3 (2.6)	5.0 (6.2)	6.0 (6.3)	1.4 (1.6)
Active days month 2^a^	24.0 (8.1)	20.8 (7.9)	21.9 (10.3)	0.6 (1.2)	3.6 (5.6)	5.0 (6.5)	0.4 (1.0)
Active days month 3^a^	22.4 (9.8)	18.9 (9.2)	20.5 (11.3)	0.3 (0.9)	3.3 (5.6)	4.5 (6.3)	0.3 (0.9)
Active days month 4^a^	20.6 (11.0)	16.9 (10.0)	19.1 (11.9)	0.1 (0.5)	2.6 (5.0)	3.8 (6.2)	0.2 (0.8)
Active days month 5^a^	19.1 (11.8)	15.4 (10.4)	17.5 (12.6)	0.1 (0.3)	2.2 (4.8)	3.4 (6.1)	0.1 (0.6)
Active days month 6^a^	18.0 (12.4)	14.9 (10.9)	16.4 (13.0)	0.1 (0.5)	2.3 (5.1)	3.5 (6.2)	0.2 (1.2)
User activity ratio^b^	0.8 (0.3)	0.7 (0.3)	0.7 (0.4)	0.0 (0.0)	0.1 (0.2)	0.2 (0.2)	0.0 (0.0)
*Intensity of engagement (mean, SD)*							
Total number of exercises/sessions	n.a.	n.a.	n.a.	7.2 (9.8)	19.0 (28.4)	30.3 (42.9)	4.3 (7.1)
Average number of daily tracked triggers	n.a.	n.a.	9.1 (2.8)	n.a.	n.a.	n.a.	n.a.
*Time (usage span) (mean, SD)*							
Stickiness^c^	141.7 (46.8)	141.6 (46.8)	132.1 (55.5)	30.8 (42.9)	83.1 (67.5)	94.4 (68.4)	43.7 (50.1)
Persistence^d^	132.6 (53.0)	129.6 (53.7)	112.6 (68.0)	4.4 (7.4)	17.8 (35.6)	24.4 (46.1)	2.5 (4.5)

Abbreviation: FITT principle, Frequency, Intensity, Time, and Type.

^a^ A month is defined as 28 days.

^b^ User Activity Ratio is calculated as total active days/ possible active days (168).

^c^ Stickiness is defined as number of days between app activation at the first study day and the last performed action.

^d^ Persistence is defined as number of days until the first longer break in app use lasting at least 7 days.

**FIGURE 3:**
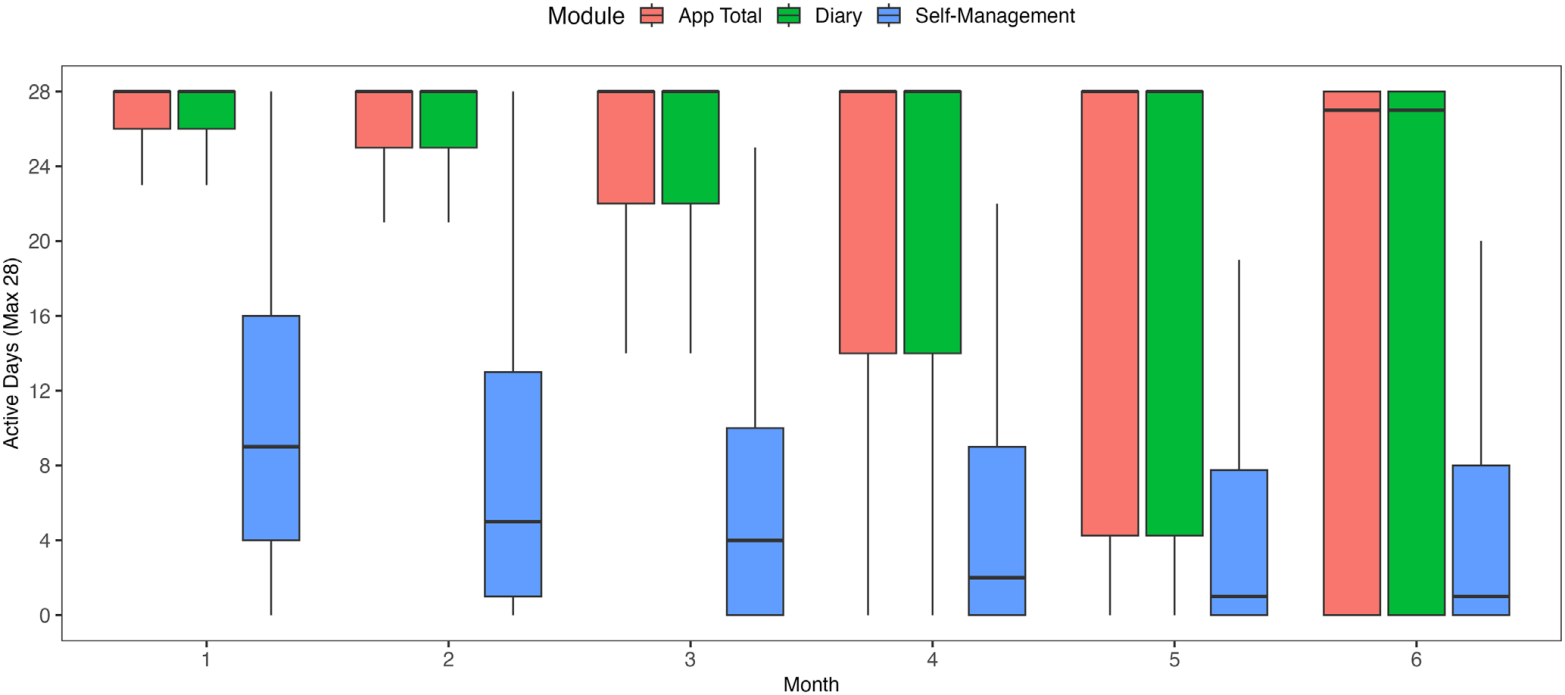
Frequency of engagement of the overall app and the features diary and “Active” self‐management, showing the number of usage days per month. [Color figure can be viewed at wileyonlinelibrary.com]

The usage frequency of the app, the diary and “Active” self‐management features is illustrated in Figure 3. The app diary was used substantially more than the self‐management feature, with a mean (SD) of 18.0 (12.4) monthly usage days after 6 months, compared to 5.0 (7.2) monthly days for the self‐management feature. Among all app modules, the trigger diary had the highest usage frequency at 6 months, with a mean (SD) of 16.4 (13.0) monthly days, followed by the headache diary with a mean (SD) of 14.9 (10.9) days per month.

The self‐management feature was not used by all participants. Among its components, the training module showed the highest engagement over the 24‐week period, with a mean (SD) of 26.2 (33.9) documented training days. In contrast, the Acute‐Help module (mean (SD) = 2.6 (4.7)) and the Education module (mean (SD) = 3.4 (4.3)) demonstrated very low total usage frequencies. Overall, participants showed limited engagement with the self‐management feature.

#### Intensity of engagement

The usage intensity of the four behavioral “Active” self‐management modules is the number of exercises/sessions completed and is shown in Table 4, and in more detail in the Supporting Information Additional File 3. Aligned with the overall engagement patterns, the training module demonstrated the highest intensity of use of the behavioral self‐management feature, with a mean (SD) of 30.3 (42.9) documented training sessions over the 168‐day study period. On average, participants completed 7.2 (SD = 9.8) knowledge lessons and had a mean (SD) of 4.3 (7.1) acute‐help sessions. On average, participants tracked 9 out of 12 possible trigger factors per day in the trigger diary.

#### Time (usage span)

After 12 weeks, 201 out of 238 participants (84.45%) were still actively using the app. By 24 weeks, this number had decreased to 161 participants (67.65%). The diary feature demonstrated greater long‐term engagement, with a mean (SD) usage duration of 141.7 (46.8) days, see Table 4. The average time to the first longer usage break, defined as at least 7 consecutive days without app activity, was a mean (SD) of 132.6 (53.0) days, indicating that participants generally used the app continuously over the study period.

**FIGURE 4:**
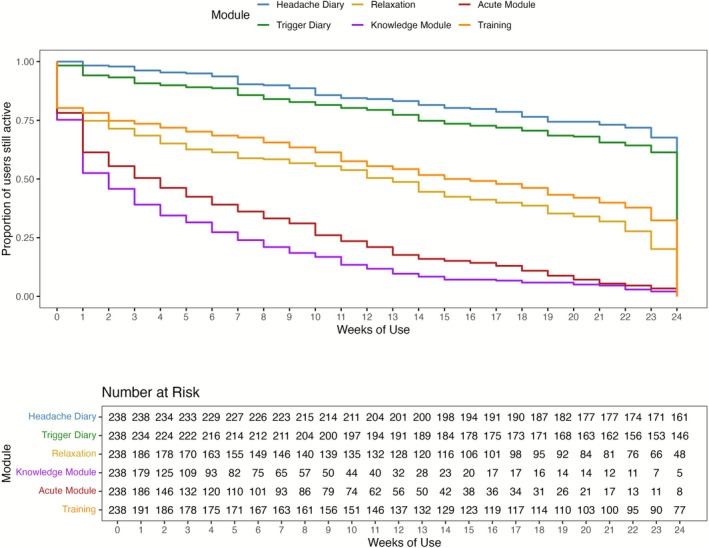
Kaplan–Meier survival curves showing the dropout of participants per app module. [Color figure can be viewed at wileyonlinelibrary.com]

Figure 4 illustrates Kaplan–Meier survival curves of the individual app modules over the 24 weeks study period. The event is defined as the last day of module usage. After 12 weeks, 201 participants (84.45%) still used the headache diary and 191 participants (80.25%) the trigger diary. The headache diary showed the highest usage stickiness, with more than half of the participants (67.65%) still using it after 24 weeks (6 months). In contrast, all four “Active” self‐management modules demonstrated faster dropout rates compared to the diary feature.

### Predictors of app usage

The results of the univariable regression analysis examining factors associated with the frequent usage of the overall app and the frequent usage of the diary and self‐management features are shown in Table 5.

**TABLE 5:** Results of univariable linear regression analyses examining factors associated with migraine app usage: Three models for overall app use, diary use, and “Active” self‐management use.

Factors	Total active days overall app use		Total active days diary use		Total active days “Active” self‐management use	
	*B* (95% CI)	*p*‐Value	*B* (95% CI)	*p*‐Value	*B* (95% CI)	*p*‐Value
*Sociodemographic*						
Age	0.85 (0.21 to 1.49)	0.01	0.85 (0.21 to 1.49)	0.01	1.12 (0.64 to 1.6)	<0.001
Gender (reference = Woman)						
Man	−4.89 (−26 to 16.21)	0.648	−4.84 (−25.96 to 16.27)	0.652	−10.83 (−27 to 5.34)	0.188
Education (reference = High School diploma with university readiness)						
High school diploma	3.96 (−14.74 to 22.67)	0.677	4.06 (−14.64 to 22.77)	0.669	9.55 (−4.76 to 23.87)	0.19
Technical college degree	−37.5 (−90.31 to 15.31)	0.163	−37.4 (−90.23 to 15.43)	0.164	−23.26 (−63.69 to 17.17)	0.258
Vocational training	−4.3 (−24.09 to 15.48)	0.669	−4.21 (−24 to 15.59)	0.676	−3.55 (−18.69 to 11.6)	0.645
Other	−2.67 (−20.14 to 14.79)	0.763	−2.59 (−20.06 to 14.88)	0.77	5.09 (−8.27 to 18.46)	0.453
*Migraine diagnosis*						
Type (reference = episodic migraine)						
Chronic migraine	5.58 (−23.88 to 35.03)	0.71	5.46 (−24.01 to 34.92)	0.716	−15.5 (−38.06 to 7.06)	0.177
Years since migraine onset	0.48 (−0.14 to 1.11)	0.131	0.48 (−0.14 to 1.11)	0.131	0.56 (0.09 to 1.04)	0.021
Years since migraine diagnosis	0.29 (−0.46 to 1.04)	0.45	0.29 (−0.46 to 1.04)	0.45	0.22 (−0.36 to 0.79)	0.459
*Burden of disease*						
HIT‐6 baseline^a^	−0.23 (−2.13 to 1.67)	0.813	−0.22 (−2.13 to 1.68)	0.817	−0.78 (−2.24 to 0.67)	0.29
HALT‐30 baseline^b^	−0.3 (−1.11 to 0.52)	0.477	−0.29 (−1.11 to 0.52)	0.481	−0.01 (−0.63 to 0.62)	0.983
HMSE‐G‐SF baseline^c^	−0.02 (−0.99 to 0.96)	0.968	−0.02 (−1 to 0.95)	0.965	0.57 (−0.17 to 1.32)	0.131
*First‐week app usage*						
Active days first week (7 days)	26.48 (20.63 to 32.33)	<0.001	26.34 (20.77 to 31.9)	<0.001	10.68 (5.87 to 15.49)	<0.001

*Note*: The predictor set included baseline patient characteristics (sociodemographic and migraine‐specific factors) as well as first‐week app usage (number of usage days in the first week).

^a^ HIT‐6 = Headache Impact Test, score ranges from 36 to 78, a lower score indicates a lower disease burden.

^b^ HALT‐30 = Headache Attributed Lost Time, score ranges from 0 to 30, a lower score indicates less lost time.

^c^ HMSE‐G‐SF = Headache Management Self‐Efficacy Scale German Short Form, score ranges from 6 to 42, a lower score indicates less self‐efficacy.

The analysis indicates that age and app usage in the first week are predictors of app engagement, both for overall app usage and for engagement with the diary and behavioral self‐management features. Older participants showed a higher usage frequency. Additionally, a higher number of app usage days in the first week was positively associated with total usage frequency.

## DISCUSSION

### Principal findings

In this secondary analysis of the EMMA trial, we examined migraine app engagement over 24 weeks and predictors of app usage. Overall, app usage was high, with a mean of 129.7 usage days over the 168‐day study period. A total of 84.5% (201/238) of participants were still actively engaged after 12 weeks, and 67.7% (161/238) remained active after 24 weeks.

The diary feature was used more frequently and for a longer duration compared to the behavioral self‐management feature, where engagement was limited. Among the behavioral self‐management modules, the training module had the highest usage frequency, with an average of 26.22 (SD 33.87) documented training days, and 32.35% (77/238) of participants remained active after 24 weeks. Usage frequency and intensity of both the education and acute‐help modules were very low.

### Interpretation

The results demonstrate very high engagement with the self‐monitoring features of the app over 6 months. Although usage frequency decreased over time, it remained at a relatively high level. Few previous studies have also shown good adherence to app‐based trigger and headache diaries; however, the decline in diary use over time was less pronounced in our study compared to others.^26^, ^40^ This may be explained by the large number of incorporated app BCTs, such as reminders, personalized feedback for daily tracking, rewards, visual data presentation, and individualized data analysis, factors that have previously been identified as predictors of app usage in other disease management apps.^47^

The app investigated in this study demonstrates that continuous, real‐time documentation of headaches and trigger factors is feasible, thereby allowing for a detailed clinical picture to be captured. Although the app included only basic descriptive headache and trigger analysis, future migraine apps may even integrate advanced analytical methods, such as machine learning, to enable headache attack prediction.^48^

Engagement with the behavioral self‐management modules was considerably lower than originally intended by the app developers and as recommended within the app itself. This finding aligns with studies on other chronic diseases, which also report high drop‐out rates for app‐based disease self‐management interventions.^38^ A large‐scale analysis of 59 mental health Android apps showed significantly higher retention rates for tracking apps than exercise apps. Self‐management interventions appear to be more difficult to integrate into daily life, even when BCTs are embedded within the app. Although the content of the education module was tailored to participants' diary entries and supported by visual illustrations, on average, only 7 out of 33 lessons were completed. This low completion rate may be due to the chatbot dialogue format, which might not have been well‐suited for delivering migraine‐specific educational content. Additionally, the lessons may have been too long or demanding.

Low usage of the relaxation and training modules may also be explained by the free‐choice approach of the self‐management component. Users had to decide for themselves which strategies to try and whether or not to set personal goals. Although the chatbot provided information about the clinical importance of these strategies and delivered personalized feedback, it did not suggest which specific strategy to start with. Given that the app included a broad range of self‐management options within its multimodal approach, patients may have required more structured guidance to initiate and select appropriate strategies. It is also possible that the variety of available exercises was perceived as too complex or overwhelming, which may have further hindered regular use. In general, the app collected little baseline information about users; for example, no questionnaires were administered at the start of app use. As a result, content tailoring could only occur on the basis of diary entries (e.g., acute‐help exercises were suggested when a tension‐type headache was reported). More fine‐grained tailoring, personalization, and tunneling may be necessary to better engage users.^47^, ^49^

Furthermore, purely digital migraine self‐management applications without support from healthcare professionals may be less effective. A systematic review showed that support by healthcare providers, such as comprehensive telecoaching, can positively influence adherence in disease management apps for cancer and respiratory diseases.^47^ In the future, hybrid care models for migraine may gain greater importance.^25^ In particular, for multimodal digital interventions, healthcare providers could discuss module use with patients or even explicitly select modules for them via a back‐end access option.

The exploratory analysis of engagement predictors showed that older age and higher app usage during the first week were associated with more frequent app usage. Other studies also have provided initial evidence that, for apps targeting headache self‐monitoring or chronic disease management, engagement increases with older age.^20^, ^47^, ^50^ However, more studies are needed to better identify engagement predictors. If app usage during the first week proves to be a general predictor, migraine app providers could consider offering short free trial periods, enabling patients to assess the app's suitability before committing to purchase or obtaining it on prescription.

The present findings are particularly relevant in light of the null results of the original EMMA trial, which showed that the full app version did not reduce migraine days more effectively than a simplified control app version containing only a headache diary^40^. Although a dose–response relationship was not examined in this secondary analysis, it is plausible that the limited use of the behavioral self‐management modules contributed to the lack of superiority of the full version. In particular, the relaxation exercises and the education module were scarcely used. Even though the training module showed the highest engagement rates among the behavioral features, the average number of endurance training days per month remained consistently below current clinical recommendations for aerobic exercise or endurance sports for migraine.^3^

## LIMITATIONS

Some limitations must be acknowledged. The EMMA trial was primarily designed to evaluate effectiveness rather than engagement. Engagement with the app's analysis feature, motivational text messages, push notifications, and instructional materials within the self‐management module was not tracked by the app. For the behavioral self‐management components, only completed and not started knowledge lessons were recorded. Another limitation of our analysis is that the trial was conducted during the coronavirus disease 2019 (COVID‐19) pandemic, and at the time of the trial phase in 2021 and 2022, due to school closures, children often had to be cared for at home in Germany. As the majority of our participants were women aged 25–45 years, these circumstances may have limited their ability to engage in relaxation exercises or training programs.

Finally, our analysis may have underestimated engagement with the behavioral self‐management components, as it focused only on micro‐level engagement, meaning interaction with the app itself, and not on macro‐level behavioral engagement.^51^ It is possible that participants performed training or relaxation exercises without documenting them in the app. Our analysis only captured activities that were logged within the app. In addition, the patient population in this study showed a high disease burden and an average migraine duration of 17.4 years. It is possible that some participants had already established routines for non‐pharmacological interventions prior to the study. However, this aspect was not assessed within the EMMA trial.

Although many hopes are placed on migraine apps to increase adherence to headache tracking and behavioral migraine self‐management, prior to this analysis, no other study had examined engagement with a comprehensive self‐management app for migraine. We demonstrate overall high engagement with the migraine app, particularly with its tracking modules, but low engagement with the behavioral self‐management modules.

To support adherence to headache self‐management apps, we suggest that future interventions adopt a more guided and tailored approach, providing concrete recommendations for specific exercises, as tailoring and personalization appear to be more effective than a purely free‐choice format.^47^, ^49^ In addition, hybrid approaches that involve healthcare providers or mentors could further enhance adherence.^52^ The integration of gamification elements, reminders, and push notifications has proven effective for promoting diary adherence in the present and in other studies, and should therefore be considered essential components of future migraine self‐management apps.^47^

## CONCLUSION

This secondary analysis demonstrated high overall engagement with a migraine app. Particularly, engagement with the diary feature remained consistently high over the 6‐month trial period, also in comparison to other disease self‐management apps. These findings support the assumption that electronic headache and trigger diaries can improve adherence to tracking and thereby enhance data quality. However, engagement with the behavioral self‐management modules was lower than intended. This highlights the ongoing challenge of achieving adherence to behavioral migraine interventions, even when supported by digital behavior change techniques.

## AUTHOR CONTRIBUTIONS

**Stefanie Lysk:** Conceptualization; writing – original draft; methodology; visualization; writing – review and editing; formal analysis; project administration; data curation; software. **Daniel Pach:** Conceptualization; writing – review and editing; methodology. **Tatjana Tissen‐Diabaté:** Methodology; formal analysis; writing – review and editing; data curation. **Simon Scholler:** Software; formal analysis; data curation; writing – review and editing. **Claudia M. Witt:** Conceptualization; methodology; supervision; writing – review and editing.

## FUNDING INFORMATION

The EMMA trial was financially supported and conducted in cooperation with the app‐developing company Newsenselab GmbH, which served as the sponsor.

## CONFLICT OF INTEREST STATEMENT

**Claudia M. Witt** received research grants to the University from the app developer Newsenselab GmbH during the conduct of the trial, and from the DIZH, Swiss Cancer Research, and the German Health Care Innovation Fund, and received personal fees from Swiss Hospitals for scientific presentations on digital health and artificial intelligence outside this work. **Daniel Pach** received grants from the German Innovation Fund outside this work. **Stefanie Lysk, Simon Scholler**, and **Tatjana Tissen‐Diabaté** declare that they have no conflicts of interest.

## Supporting information


**Appendix S1:** Supporting Information.

**Appendix S2:** Supporting Information.

**Appendix S3:** Supporting Information.
