# Supplementary material for: Engagement and predictors of use of a smartphone app for migraine self‐management: A secondary analysis of the EMMA trial
Source: Headache. 2025 Nov 25;66(1):118–31. doi: 10.1111/head.70009 (PMC12849534; doi:10.1111/head.70009)
Supplement: Supplementary file 1 — Appendix S1: Supporting Information. [file HEAD-66-118-s003.docx]

Additional File 1 – List of Behavior Change Techniques

**Table 1.** List of Behavior Change Techniques (BCTs) integrated in the app, according to BCT- Ontology by Marques [1]

| **BCT group** | **BCT Ontology - ID** | **BCT** |
| --- | --- | --- |
| Goal directed BCT | BCIO:007010 | Action planning BCT |
|  | BCIO:007016 | Advise goal integration |
|  | BCIO:007015 | affirm commitment BCT |
|  | BCIO:007004 | agree behavior goal BCT |
|  | BCIO:007003 | set behavior goal |
| Advise to seek support BCT | BCIO:007033 | advise to seek appraisal support BCT |
|  | BCIO:007030 | advise to seek instrumental support BCT |
| Monitoring BCT | BCIO:007023 | provide feedback on behavior |
|  | BCIO:007027 | provide feedback on outcome of behavior |
|  | BCIO:007019 | Record behavior without feedback |
|  | BCIO:007024 | self-monitor behavior |
|  | BCIO:007025 | self-monitor outcome of behavior |
| Alter external stimulus BCT | BCIO:007080 | prompt intended action |
| Guide how to perform behavior BCT | BCIO:007051 | agree on how to perform behavior BCT |
|  | BCIO:007058 | instruct how to perform a behavior BCT |
|  | BCIO:007303 | suggest how to perform behavior |
| Increase awareness of consequences BCT | BCIO:007119 | imagine reward BCT |
|  | BCIO:007063 | inform about health consequences BCT |
| Advise specific behavior BCT | BCIO:007094 | practise behavior BCT |
| Promote pharmacological support BCT | BCIO:007146 | encourage pharmacological support BCT |
| Prompt thinking related to successful performance BCT | BCIO:007137 | persuade about personal capability |
|  | BCIO:007139 | prompt focus on past success |
|  | BCIO:007060 | remind about personal capability |

Reference

1. Marques MM, Wright AJ, Corker E, Johnston M, West R, Hastings J, Zhang L, Michie S (2023) The Behavior Change Technique Ontology: Transforming the Behavior Change Technique Taxonomy v1. Wellcome Open Res 8:308. doi:10.12688/wellcomeopenres.19363.1
