# Supplementary material for: Engagement and predictors of use of a smartphone app for migraine self‐management: A secondary analysis of the EMMA trial
Source: Headache. 2025 Nov 25;66(1):118–31. doi: 10.1111/head.70009 (PMC12849534; doi:10.1111/head.70009)
Supplement: Supplementary file 2 — Appendix S2: Supporting Information. [file HEAD-66-118-s001.pdf]

2nd ANNEX 21 March 2025

This annex adds an exploratory analysis of user engagement to the SAP version 2.1 of the EMMA study (DRKS00024174).

SPECIFICATION

User engagement was previously addressed in the SAP under Section 6.3, "*Further measurements*" (Tracked Usage of App Features). However, we would like to add a more detailed post-hoc analysis. Additionally, the original analysis was limited to a 12-week period, whereas we aim to include an analysis of usage data throughout the 12 week follow up study. Furthermore, we intend to identify predictors for the frequency of app usage.

APP MODULES AND ENGAGEMENT OUTCOMES

- For the intervention group, the usage data of the entire app, as well as the app features "Diary" and "Self-Care" and the respective associated modules, will be examined over time. The app-modules are listed under Section 6.3. However, the list has two mistakes. The app did not log the usage of the Analysis feature and the Physician report. Additionally, the medication intake diary was part of the headache reporting. We have corrected the list of the app-modules as follows:
    - (a) headache diary
    - (b) Trigger factor diary
    - (c) Knowledge module
    - (d) Relaxation module
    - (e) Training module
    - (e) Acute help module
- App engagement will be assessed based on the FITT model (Frequency, Intensity, Time, Type) (Short et al., 2018) by calculating usage frequency, usage intensity, and usage duration. Tables 1–3 present all newly derived outcomes for measuring user engagement, categorized according to the FITT model. All measurements are taken daily. However, for deriving the outcomes, data is aggregated across months (one month is defined as 28 days). All resulting scales are count variables but are treated as continuous. Higher values indicate greater engagement/usage.

Table 1. New outcomes for measuring usage <u>frequency</u>

Metric	Outcomes	Definition	Time frame	Possible values
Total usage frequency overall app	Total active days app	Number of days with app usage over the 24 weeks	Week 1-24	0-168
Monthly usage frequency of the overall app	Monthly active days app, month 1-6	Number of days with app usage per month, during months 1-6	monthly	0-28
Usage frequency of the overall app first week	Active days app week 1	Number of days with app usage during the first week	Week 1	0-7
Total usage frequency of app features	Total active days diary	Number of days with diary usage over the 24 weeks	Week 1-24	0-168
	Total active days self-care	Number of days with entries or completed exercises in the self-care feature of the app over the 24 weeks	Week 1-24	0-168
Monthly usage frequency of app features	Monthly active days diary, month 1-6	Number of days with diary usage per month, during months 1-6	monthly	0-28
	Monthly active days self-care, month1-6	Number of days with entries or completed exercises in the self-care section of the app, during month 1-6	monthly	0-28
Total usage frequency of the app modules	Total active days Headache diary	Number of days with headache diary entries over the 24 weeks	Week 1-24	0-168
	Total active days Trigger diary	Number of days with trigger diary entries over the 24 weeks	Week 1-24	0-168
	Total active days Relaxation module	Number of days with relaxation exercises over the 24 weeks	Week 1-24	0-168
	Total active days Training module	Number of days with training sessions over the 24 weeks	Week 1-24	0-168
	Total active days Knowledge module	Number of days with completed knowledge lessons over the 24 weeks	Week 1-24	0-168
	Total active days Acute module	Number of days with acute help exercises over the 24 weeks	Week 1-24	0-168
Monthly usage frequency of the app modules	Monthly active days Headache diary, month 1-6	Number of days with headache diary entries per month	monthly	0-28
	Monthly active days Trigger diary, month 1-6	Number of days with trigger diary entries per month	monthly	0-28
	Monthly active days Relaxation module, month 1-6	Number of days with relaxation exercises per month	monthly	0-28
	Monthly active days Training module, month 1-6	Number of days with training sessions per month	monthly	0-28
	Monthly active days Knowledge module, month 1-6	Number of days with completed knowledge lessons per month	monthly	0-28
	Monthly active days Acute module, month 1-6	Number of days with acute help exercises per month	monthly	0-28
User Activity Ratio	User Activity Ratio App	Number of "total active days app" divided by the total number of days in the study (168)	Week 1-24	0-1
	User Activity Ratio Diary	Number of "total active days diary" divided by the total number of days in the study (168)	Week 1-24	0-1
	User Activity Ratio Self-Care	Number of "total active days self-care" divided by the total number of days in the study (168)	Week 1-24	0-1

Table 2. New outcomes for measuring usage intensity

Metric	Outcomes	Definition	Time frame	Possible values
Usage intensity of trigger factor diary	Average number tracked trigger	Average number of tracked trigger factors over the 24 weeks (max. 12 factors); based on days with trigger diary entries; days without entries are excluded from the calculation	week 1-24	0-12
Usage intensity of knowledge module	Total number knowledge lessons	Total number of knowledge lessons completed over 24 weeks	week 1-24	0-33
Usage intensity of relaxation module	Total number of relaxation exercises	Total number of relaxation exercises performed over 24 weeks	week 1-24	≥0
Usage intensity of training module	Total number of training sessions	Total number of documented endurance training sessions over 24 weeks	week 1-24	≥0
Usage intensity of acute help module	Total number of acute help exercises	Total number of exercises performed in the acute help module over 24 weeks	week 1-24	≥0

Table 3. New outcomes for measuring usage duration

Metric	Outcome	Definition	Time frame	Possible values
Total time of using the app	Stickiness app	Number of days from app activation to the last day of app use	week 1-24	0-168
	Persistence app	Number of days until the first longer break in app use lasting at least 7 days	week 1-24	0-168
Total time of using app features	Stickiness Diary	Number of days between app activation and the last app diary entry	week 1-24	0-168
	Persistence Diary	Number of days until the first longer break without diary entries for at least 7 days	week 1-24	0-168
	Stickiness Self-Care	Number of days between app activation and the last entry or last exercise in the self-care module	week 1-24	0-168
	Persistence Self-Care	Number of days until the first longer break without the use of the self-care feature lasting at least 7 days	week 1-24	0-168
Total time of using app modules	Stickiness headache diary	Number of days between app activation and the last headache diary entry	week 1-24	0-168
	Stickiness trigger diary	Number of days between app activation and the last trigger diary entry	week 1-24	0-168
	Stickiness relaxation module	Number of days from app activation to the last performed relaxation exercise	week 1-24	0-168

	Stickiness training module	Number of days between app activation and the last performed endurance training session	week 1-24	0-168
	Stickiness knowledge module	Number of days between app activation and the last completed knowledge lesson	week 1-24	0-168
	Stickiness acute help module	Number of days between app activation and the last performed exercise in the acute module	week 1-24	0-168

ADDITIONAL POST-HOC ENGAGEMENT ANALYSIS

Missing Data

- The newly derived variables by definition do not have missing values, as they are initially calculated daily with values of 0 (no entry/no usage) or 1 (usage). Even when calculating intensity outcomes, e.g. the number of completed exercise, the dataset contains no missing values. On days without entries or app logs, the value for that day is set to 0. Only in cases where a user drops out of the study is their engagement data set to missing after dropout. Subsequently, usage frequency and usage intensity are calculated for the reduced study time only.

Statistical Analysis

- To analyze user engagement over time, descriptive analyses (means and standard deviations, minimum and maximum values, medians and interquartile ranges) will be calculated for all outcome variables (usage frequency, intensity and duration) from Tables 1-3. Additionally, a visual inspection of the data distribution will be performed. To visualize the data, engagement curves (based on means with standard deviations) will be presented for the entire app, both features and each module over the 24-week usage period.
- To analyse usage duration, metrics such as stickiness and persistence will be calculated and visualized using survival curves (Kaplan-Meier curves).
- To examine engagement predictors, the frequency of app usage will be operationalized using the variable "total active days app", “total active days diary” and “total active days self-care”. All patient characteristics (baseline variables) and app usage frequency during the first week ("Active days app week 1"), will be analyzed as predictors. Multiple linear regression analyses will be conducted, with "Total active days app", “Total active days diary” or “Total active days self-care” as the dependent variables.
- Stefanie Lysk will perform for the post hoc engagement analysis.

REFERENCE

Short, C. E., DeSmet, A., Woods, C., Williams, S. L., Maher, C., Middelweerd, A., Muller, A. M., Wark, P. A., Vandelanotte, C., Poppe, L., Hingle, M. D., & Crutzen, R. (2018). Measuring Engagement in eHealth and mHealth Behavior Change Interventions: Viewpoint of Methodologies. *J Med Internet Res*, *20*(11), e292. https://doi.org/10.2196/jmir.9397

Principal investigator

____

Claudia M. Witt, MD, MBA

Co-Principal investigator

____

Daniel Pach, MD

Data management & Statistics

____

Stefanie Lysk

Statistician

____

Tatjana Tissen-Diabaté
