# Supplementary material for: Engagement and predictors of use of a smartphone app for migraine self‐management: A secondary analysis of the EMMA trial
Source: Headache. 2025 Nov 25;66(1):118–31. doi: 10.1111/head.70009 (PMC12849534; doi:10.1111/head.70009)
Supplement: Supplementary file 3 — Appendix S3: Supporting Information. [file HEAD-66-118-s002.docx]

**Table 1.** Descriptive analysis of the frequency of engagement for the overall app and the diary feature

|  | **Overall app** | | | **Diary Feature** | | | **Headache diary module** | | | | **Trigger diary module** | | |
| --- | --- | --- | --- | --- | --- | --- | --- | --- | --- | --- | --- | --- | --- |
|  | Mean (SD) | Median (IQR) | Min-Max | Mean (SD) | Median (IQR) | Min-Max | Mean (SD) | | Median (IQR) | Min-Max | Mean (SD) | Median (IQR) | Min-Max |
| **Frequency of engagement** |  | | | | | | | | | | | | |
| Total active days over 24 weeks | 129.68 (52.48) | 162.5 (70) | 1-168 | 129.64 (52.51) | 162.5 (70) | 1-168 | 110.56 (49.72) | 128 (77.75) | | 1-168 | 118.12 (60.94) | 156 (98) | 0-168 |
| User activity ratio | 0.78 (0.31) | 0.96 (0.42) | 0.01-1 | 0.77 (0.31) | 0.97 (0.42) | 0.01-1 | 0.66 (0.30) | 0.76(0.46) | | 0.01-1 | 0.70 (0.36) | 0.92 (0.58) | 0-1 |
| Active days month 1 | 25.63 (5.32) | 28 (2) | 1-28 | 25.61 (5.33) | 28 (2) | 1-28 | 23.64 (5.54) | 25 (4) | | 1-28 | 22.63 (8.85) | 28 (7.75) | 0-28 |
| Active days month 2 | 24.01 (8.09) | 28 (3) | 0-28 | 24.00 (8.09) | 28 (3) | 0-28 | 20.84 (7.90) | 24 (9) | | 0-28 | 21.94 (10.25) | 28 (9) | 0-28 |
| Active days month 3 | 22.40 (9.82) | 28 (6) | 0-28 | 22.38 (9.83) | 28 (6) | 0-28 | 18.86 (9.23) | 22 (11) | | 0-28 | 20.54 (11.28) | 28 (15) | 0-28 |
| Active days month 4 | 20.62 (11.00) | 28 (14) | 0-28 | 20.62 (11.01) | 28 (14) | 0-28 | 16.94 (9.96) | 20 (17.50) | | 0-28 | 19.12 (11.90) | 28 (23) | 0-28 |
| Active days month 5 | 19.05 (11.82) | 28 (23.75) | 0-28 | 19.05 (11.82) | 28 (23.75) | 0-28 | 15.40 (10.44) | 19 (20.75) | | 0-28 | 17.52 (12.57) | 27.5 (28) | 0-28 |
| Active days month 6 | 17.98 (12.44) | 27 (28) | 0-28 | 17.98 (12.44) | 27 (28) | 0-28 | 14.89 (10.94) | 19 (25) | | 0-28 | 16.37 (13.01) | 27 (28) | 0-28 |
| **Time (usage span)** |  | | | | | | | | | | | | |
| Stickiness^a^ | 141.72 (46.83) | 168 (37) | 1-168 | 141.72 (46.83) | 168 (37) | 1-168 | 141.72 (46.83) | 168 (37) | | 1-168 | 132.11 (55.46) | 168 (69.5) | 0-168 |
| Persistence^b^ | 132.55 (52.99) | 168 (67.75) | 1-168 | 132.09 (53.16) | 168 (69.5) | 1-168 | 132.09 (53.16) | 168 (69.5) | | 1-168 | 112.61 (67.95) | 168(123.5) | 0-168 |

a Stickiness = number of days between app activation and the last performed action

b Persistence = number of days until the first longer break in app use lasting at least seven days

**Table 2.** Descriptive analysis of the frequency and intensity of engagement for the self-management feature

|  | **Self-management feature** | | | **Relaxation module** | | | **Training module** | | | **Education module** | | | **Acute-help module** | | |
| --- | --- | --- | --- | --- | --- | --- | --- | --- | --- | --- | --- | --- | --- | --- | --- |
|  | Mean (SD) | Median (IQR) | Min-Max | Mean (SD) | Median (IQR) | Min-Max | Mean (SD) | Median (IQR) | Min-Max | Mean (SD) | Median (IQR) | Min-Max | Mean (SD) | Median (IQR) | Min-Max |
| **Frequency of engagement** | | | | | | | | | | | | | | | |
| Total active days over 24 weeks | 40.05 (40.13) | 27 (50) | 0-165 | 19.00 (28.42) | 6 (24.75) | 0-164 | 26.22 (33.87) | 13 (34.75) | 1-153 | 3.39 (4.29) | 2 (4) | 0-26 | 2.58 (4.69) | 2 (2) | 0-59 |
| User activity ratio | 0.24 (0.24) | 0.16 (0.30) | 0-0.92 | 0.11 (0.17) | 0.04 (0.15) | 0-0.98 | 0.16 (0.20) | 0.08 (0.21) | 0-0.91 | 0.02 (0.03) | 0.01 (0.02) | 0-0.15 | 0.02 (0.03) | 0.01 (0.01) | 0-0.35 |
| Active days month 1 | 10.64 (7.86) | 9 (12) | 0-28 | 4.96 (6.22) | 2 (8) | 0-28 | 5.98 (6.28) | 4 (9.75) | 0-27 | 2.27 (2.57) | 1 (3) | 0-11 | 1.37 (1.64) | 1 (2) | 0-10 |
| Active days month 2 | 7.68 (7.92) | 5 (12) | 0-28 | 3.58 (5.64) | 1 (5) | 0-28 | 5.03 (6.53) | 2 (7) | 0-27 | 0.57 (1.17) | 0 (1) | 0-8 | 0.42 (0.95) | 0 (1) | 0-9 |
| Active days month 3 | 6.61 (7.63) | 4 (10) | 0-28 | 3.29 (5.56) | 0 (4) | 0-27 | 4.48 (6.29) | 1 (7) | 0-27 | 0.30 (0.88) | 0 (0) | 0-6 | 0.30 (0.93) | 0 (0) | 0-10 |
| Active days month 4 | 5.49 (7.26) | 2 (9) | 0-28 | 2.59 (5.04) | 0 (3) | 0-28 | 3.80 (6.18) | 0 (5.75) | 0-28 | 0.12 (0.47) | 0 (0) | 0-4 | 0.20 (0.76) | 0 (0) | 0-9 |
| Active days month 5 | 4.66 (6.92) | 1 (7.75) | 0-28 | 2.21 (4.87) | 0 (1.75) | 0-28 | 3.42 (6.05) | 0 (4) | 0-28 | 0.05 (0.25) | 0 (0) | 0-2 | 0.14 (0.57) | 0 (0) | 0-6 |
| Active days month 6 | 4.97 (7.24) | 1 (8) | 0-28 | 2.32 (5.06) | 0 (2) | 0-28 | 3.51 (6.21) | 0 (4.75) | 0-28 | 0.08 (0.48) | 0 (0) | 0-6 | 0.15 (1.19) | 0 (0) | 0-18 |
| **Intensity of engagement** | | | | | | | | | | | | | | | |
| Total number of sessions/lessons | n.a. | n.a. | n.a. | 19.00 (28.42) | 6 (24.75) | 0-164 | 30.33 (42.86) | 13 (37.75) | 0-281 | 7.16 (9.84) | 3(7) | 0-63 | 4.33 (7.06) | 2 (5) | 0-84 |
| **Time (usage span)** | | | | | | | | | | | | | | | |
| Stickiness^a^ | 110.75 (62.19) | 144 (109) | 0-168 | 83. 13 (67.53) | 85 (149.75) | 0-168 | 94.44 (68.35) | 107.5 (149.75) | 0-168 | 30.82 (42.87) | 10 (43.75) | 0-168 | 43.68 (50.06) | 22 (71.75) | 0-168 |
| Persistence^b^ | 40.91 (52.76) | 17 (50.25) | 0-168 | 17.83(35.55) | 2(17) | 0-168 | 24.35 (46.13) | 3(21.75) | 0-168 | 4.42(7.44) | 1.5(6) | 0-61 | 2.49(4.46) | 0(3.75) | 0-31 |

a Stickiness = number of days between app activation and the last performed action

b Persistence = number of days until the first longer break in app use lasting at least seven days


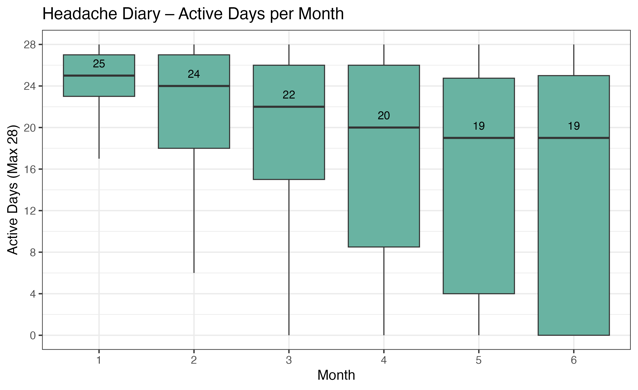

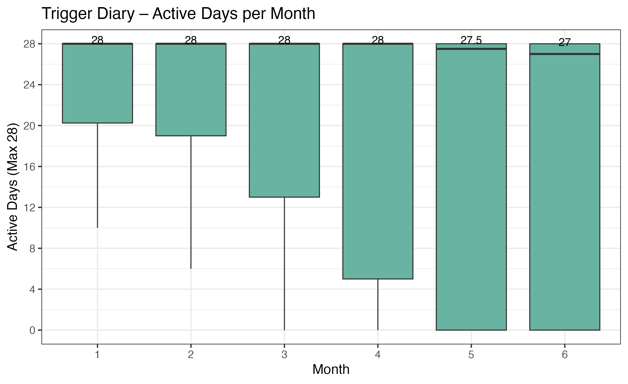


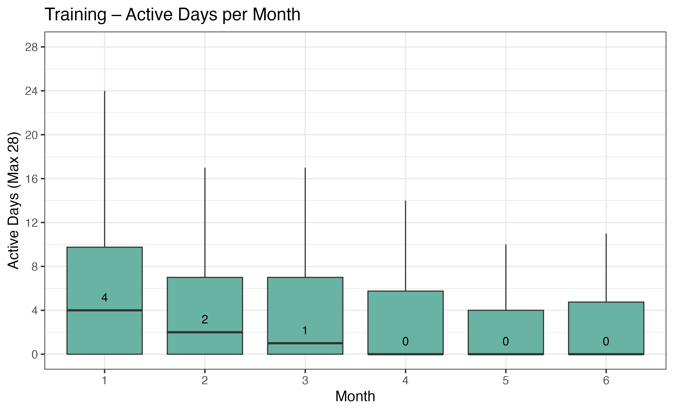

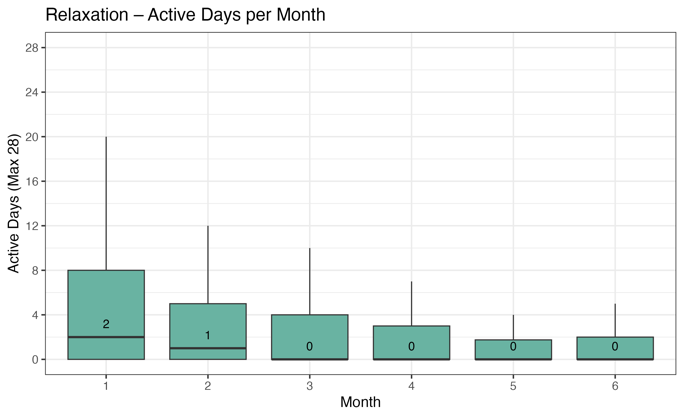


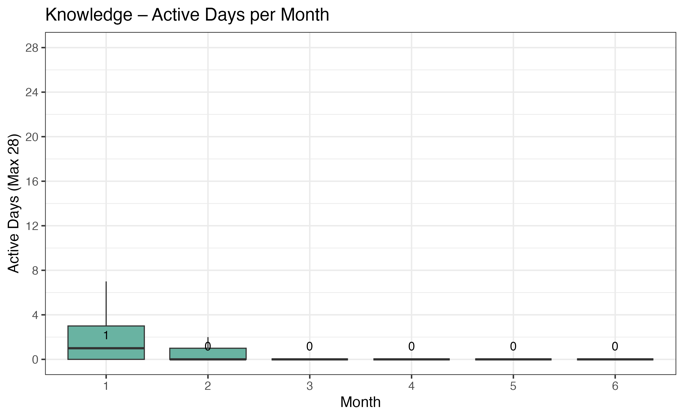

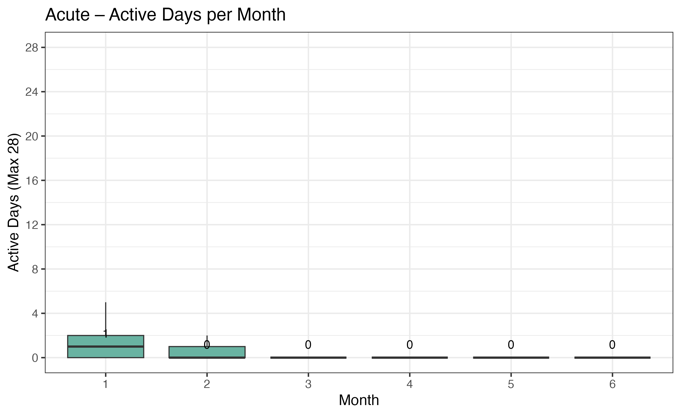


**Fig. 1** Monthly frequency of app engagement (number of usage days) across six modules (headache diary, trigger diary, training, relaxation, knowledge, acute module); medians are indicated by horizontal lines within each boxplot.


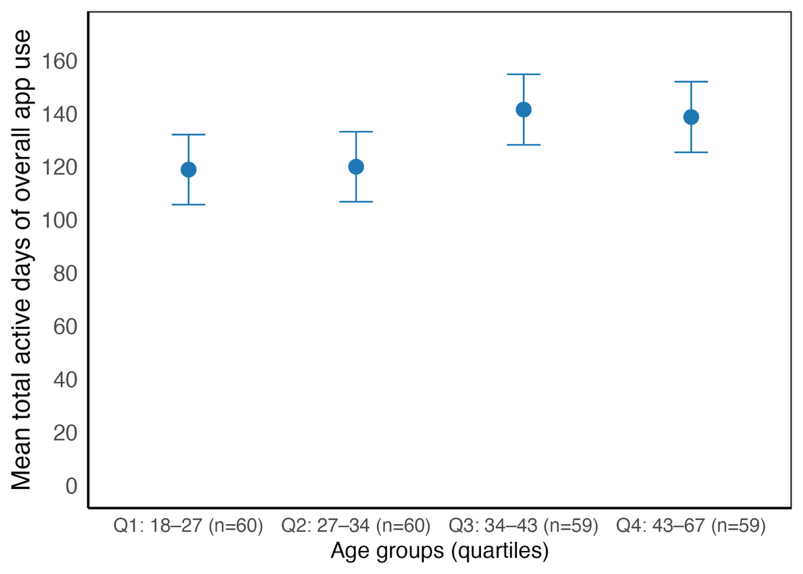

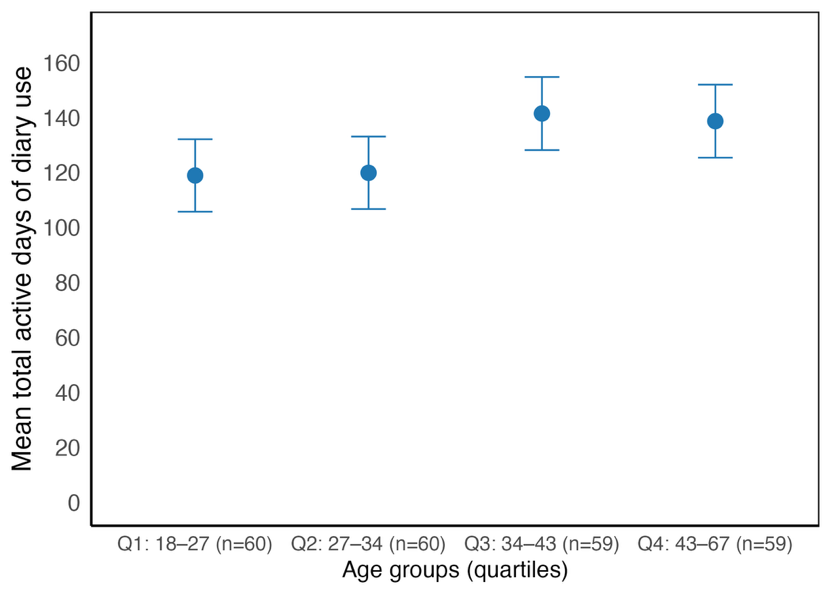


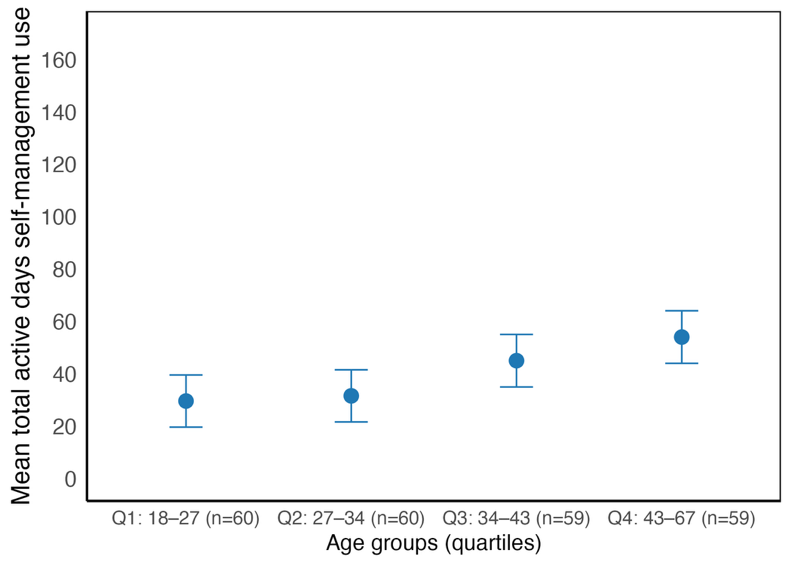


**Fig. 2** Mean total active days of overall app use, diary use, and self-management use across age quartiles (shown as means ± 95% confidence interval)


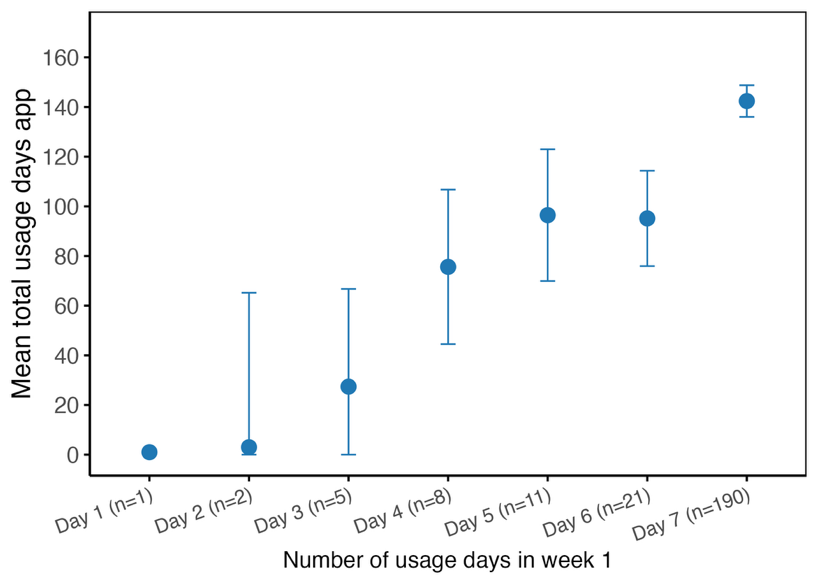

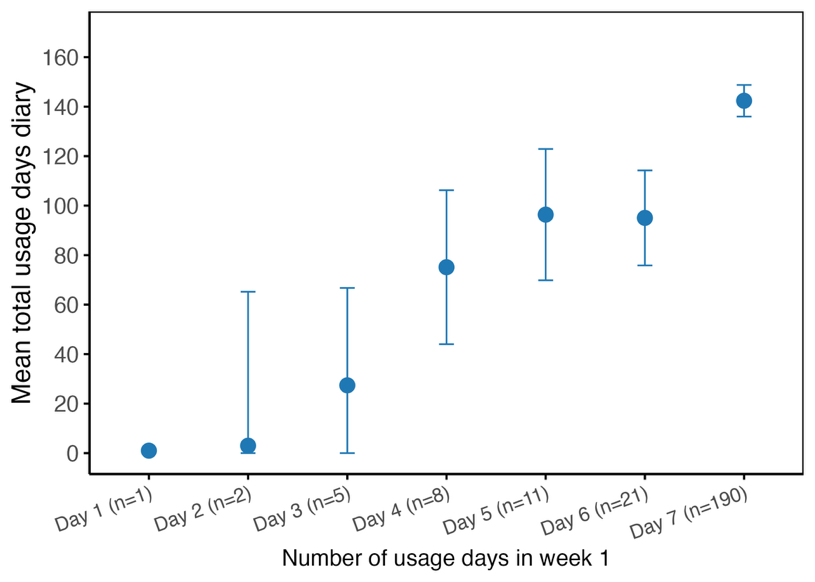


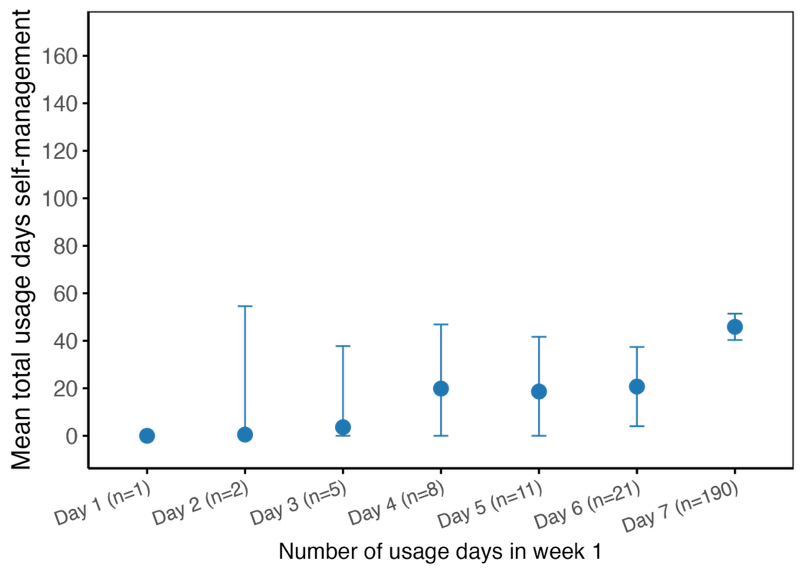


**Fig. 3** Mean total active days of overall app use, diary use, and self-management use across number of app usage days in week 1 (shown as means ± 95% confidence interval)
